# Progress on Infrared Imaging Technology in Animal Production: A Review

**DOI:** 10.3390/s22030705

**Published:** 2022-01-18

**Authors:** Shuailong Zheng, Changfan Zhou, Xunping Jiang, Jingshu Huang, Dequan Xu

**Affiliations:** 1 Key Laboratory of Swine Genetics and Breeding, Ministry of Agriculture and Rural Affairs, Huazhong Agricultural University, Wuhan 430070, China; zhengshuailong1996@163.com (S.Z.); zhouchangfan1991@163.com (C.Z.); 2 Key Laboratory of Agricultural Animal Genetics, Breeding and Reproduction of Ministry of Education, Huazhong Agricultural University, Wuhan 430070, China; xpjiang@mail.hzau.edu.cn; 3 Colleges of Animal Science & Technology, Huazhong Agricultural University, Wuhan 430070, China; 4 Agricultural Development Center of Hubei Province, Wuhan 430064, China; hjs1996@163.com

**Keywords:** IRT imaging technology, influencing parameters, disease diagnosis, estrus identification, pregnancy diagnosis

## Abstract

Infrared thermography (IRT) imaging technology, as a convenient, efficient, and contactless temperature measurement technology, has been widely applied to animal production. In this review, we systematically summarized the principles and influencing parameters of IRT imaging technology. In addition, we also summed up recent advances of IRT imaging technology in monitoring the temperature of animal surfaces and core anatomical areas, diagnosing early disease and inflammation, monitoring animal stress levels, identifying estrus and ovulation, and diagnosing pregnancy and animal welfare. Finally, we made prospective forecast for future research directions, offering more theoretical references for related research in this field.

## 1. Introduction

The livestock industry is essential around the world, with livestock systems covering 30% of the earth’s land surface area and approximately 3 billion people working in livestock worldwide [1]. However, traditional animal husbandry has many problems, such as high cost, low production efficiency, and environmental pollution [2]. The new round of information technology revolution represented by the Internet of Things, cloud computing, big data and artificial intelligence promotes the transformation of roughly traditional animal husbandry to knowledge-based, technology-based, and modernized intelligent animal husbandry. In addition, technological advantages have become an essential factor driving the rapid development of animal husbandry [3,4]. Intelligent animal husbandry urgently needs new technical means to be used in animal production to achieve accurate, real-time, and dynamic monitoring of animal physiological states [5,6,7,8].

In mammals, the body temperature normally presents at 37 °C. Maintaining a relatively constant core body temperature is essential to maintaining optimal body system function and sensitive biochemical response processes [9]. Recent scientific studies have shown that a fatal brain damage might occur when the temperature is higher than 45 °C, whereas cardiac fibrillation, respiratory rate decrease, and even death might occur when temperatures are below 27–29 °C [10,11]. In addition, when animals are in a state of stress, estrus or pregnancy, or have infectious diseases, the autonomic sympathetic nerves in the body release catecholamines. Catecholamines can result in peripheral vasoconstriction and blood flow increase, causing changes in body temperature [12,13,14]. Therefore, in various physiological indicators of animals, the difference in body temperature is an essential indicator for evaluating animal physiological health and preventing diseases [15]. However, temperature assessment is often performed using invasive methods that cause stress to the animal, such as rectal thermometry [16,17]. As a fast and efficient temperature measurement technology, IRT can be converted into visualized thermal images by identifying the surface temperature of the target object [18]. Then, infrared thermal images can be analyzed using professional software, enabling a fast comparison of temperatures between target areas [19]. In addition, IRT is real-time, noninvasive, and does not expose living organisms to strong, harmful radiation like x-rays [20]. Over the past few years, IRT has been widely used in the fields of medicine [21,22,23], agriculture [24,25,26,27,28], architecture [29,30], energy [31], and aviation [32,33]. IRT imaging technology has also been applied to animal production [34]. By detecting changes in surface blood circulation without contact, IRT can detect and measure levels of thermal radiation changes in the animal body’s surface, improving animal welfare and meeting the needs of intelligent animal husbandry in the future [35,36,37]. Therefore, IRT imaging technology is considered one of the most promising technical methods in animal production [38].

In this review, we introduce the principles and parameters of IRT imaging technology in detail. In addition, we also systematically summarize the latest application of IRT imaging technology and prospect the future application of IRT imaging technology in animal production to provide a reference for the related research fields.

## 2. Overview of IRT Imaging Technology

The advantages of IRT imaging technology include its convenience, speed of collection, and noncontact method [39]. It has been commonly used to detect the surface or core temperature, track and forecast early illnesses, detect stress levels, and diagnose pregnancy. However, the realistic implementation of IRT imaging technology necessitates a thorough understanding of the technical principles of IRT imaging and the various parameters affecting IRT imaging [40].

### 2.1. Principles of IRT Imaging Technology

When studying light dispersion, F.W. Herschel observed infrared thermal influence for the first time. In nature, when an object’s surface temperature reaches absolute zero (−273.15 °C), the object can emit electromagnetic waves. There is no thermal energy emitted below absolute zero. Hence, the change in reading reaches above absolute zero [41]. Furthermore, an electromagnetic wave’s radiation strength and wavelength propagation properties vary with the weather changes. An infrared ray, known as an electromagnetic wave, has a wavelength range of 0.75~1000 μm. However, only the short wave band (1–3 μm), intermediate wave band (3–6 μm), and longwave band (6–15 μm) can spread across the atmosphere [40]. The infrared thermal imager measures the surface temperature of an object by using the object’s radiation’s infrared longwave band. According to the Stefan-Boltzmann Formula (1), the infrared radiation energy of an object is proportional to the fourth power of its temperature [40].

*W*_obj_ = *ε* × *σ* × *Τ*_obj_^4^ (1)

where *W*_obj_ is the thermal radiation emitted by an object, *Τ*_obj_ is the object’s temperature (measured in K), *ε* is the Stefan–Boltzmann constant (ca 5.67 × 10^−8^ Wm^−2^K^−4^), and *σ* is the emissivity of the object [42].

**Figure 1:**
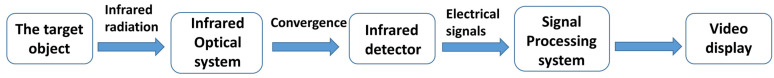
The working principle of the IRT imaging camera.

The IRT imager is a photoelectric instrument passively receiving target infrared radiation. It is composed of four parts: an optical system, an infrared detector, a signal processing machine, and a monitor (Figure 1) [43]. The infrared imager receives infrared radiation energy from the target object via the optical system’s objective lens. After concentrating, the radiation energy reaches the infrared detector used for transforming energy into electrical signals. Finally, the signal processing circuit converts the electric signals into infrared images. For animals, the radiation intensity can vary significantly, resulting in the color variation of the infrared heat map. Using this trait, multiple temperatures of the animal body surface can be obtained dynamically. Compared with other temperature measurement methods (such as thermocouples and mercury thermometers), the IRT imager has many advantages in temperature measurement [44]. To begin with, unlike a thermocouple with a single contact point, an infrared imager can easily track temperature change on one or several targets simultaneously [45,46]. Second, IRT reacts quickly to temperature changes in the target object and can detect a moving or rapidly changing object [47]. Finally, it uses a non-contact method, critical for tracking a distant animal’s body temperature [44]. Of course, the application of infrared thermography has certain limitations. Firstly, IRT is susceptible to weather conditions, such as solar radiation, wind, and rainfall, affecting the measurement’s accuracy [48,49]. Secondly, the physical properties of the animal’s epidermis, such as the thickness and quality of the fur or feather cover, significantly affect the temperature of the outer surface of the body [50]. Finally, physical activity also affects the accuracy of the measurement, as physical activity (running, training) before the measurement causes heat to be radiated by the skeletal muscles, thus increasing the temperature of the body surface [51].

### 2.2. Parameters Affecting IRT Imaging

IRT imaging is a commonly used technology in animal production. It measures the temperature of an object utilizing an IRT detector. However, some thermal imaging parameters, such as the emissivity of the target object, the reflected temperature, the distance between the IRT imager and the target, the ambient temperature, and humidity, are critical to measurement accuracy [52]. These thermal imaging parameters must be entered into the calculation to determine the target object’s temperature reliably. Furthermore, for accurate temperature acquisition, the professional use of an IRT imager and proper model selection is also critical. The choice of imaging angle of thermal imaging and the different resolutions of thermal imagers have an impact on the accuracy of the results.

#### 2.2.1. Emissivity

The emissivity of an object (ε), also known as the emissivity or emission coefficient, is a ratio between 0 and 1. It indicates an object’s ability to radiate infrared energy compared to a black body at the same temperature [19]. If the object’s emissivity under test is 1, it is a black body [53]. Additionally, the reflected temperature has little effect on objects with high emissivity. However, for objects with low emissivity, the total infrared radiation energy collected by the IRT imager is significantly influenced by the reflected temperature. Emissivity is mainly affected by the composition of the object to be measured. For instance, animals’ body surface emissivity is typically between 0.86 and 0.98 relative to other objects.

Additionally, emissivity is also affected by the object’s properties, such as the geometry and surface structure of the object [39]. The wrong selection of emissivity has the most significant influence on temperature measurement accuracy. According to the Stefan_Boltzmann formula, the amount of radiation energy received by the IRT imager is proportional to the object’s emissivity. Therefore, to obtain accurate measurement results, the emissivity of the input IRT imager should be compatible with the measured object; otherwise, temperature measurement results would deviate. The emissivity value for an animal is almost always listed as 0.98 irrespective of species [54,55].

#### 2.2.2. Reflected Temperature

Reflected temperature estimates the level of background radiation reflected from a thermal imaging object and is usually shown as a temperature value [19]. The reflected temperature is also known as “apparent reflected temperature”, “background radiation”, and “reflected radiation from environmental sources”. In nature, unlike black bodies, objects are influenced by the reflected temperature; that is, a portion of the total radiation reaching the thermal imaging equipment is produced by the background environment itself [56]. Since the temperature in most conditions is not uniform, measuring the reflected temperature accurately is difficult. In specific applications, particularly indoors, air temperature is typically treated as reflected temperature. In outdoor applications, sky temperature is more appropriate, but it is affected by cloud cover and different day periods [19]. In fact, due to the high emissivity of biological tissue, the influence of reflected temperature on infrared temperature measurement is relatively low [20].

#### 2.2.3. Ambient Temperature, Humidity, and Measurement Distance

Other than emissivity and reflection temperature, some factors such as ambient temperature, and humidity, along with the distance between the IRT imager and the target object are also significant factors affecting the accuracy of IRT imaging [19,39]. Assuming the target sample had a high ambient temperature, such as being under direct sunlight or surrounded by high-temperature objects, the surface temperature would increase, resulting in an IRT measurement error [57]. Furthermore, when an object is exposed to the sun’s rays, it can reflect solar radiation to the IRT imager and impact temperature measurement accuracy [58].

The humidity of the area and the distance (d) between the camera and the target object can also affect the precision of the temperature measurement by affecting infrared transmittance in the atmosphere. Many gas molecules and suspended particles in the atmosphere, including water vapor, carbon dioxide, ozone, smoke, and bacteria, strongly absorb infrared radiation. They also have a particular scattering effect on infrared radiation [40]. When the number of gas molecules and suspended particles in the air increases, so does their absorption and scattering effect, resulting in a decrease in infrared transmittance in the atmosphere. Of course, with the rise in humidity in the object’s environment, the water vapor molecules in the background would increase, reducing the infrared transmission [59]. In that case, atmospheric humidity is crucial to the accuracy of infrared temperature measurement. In addition, as the IRT imager moves farther away from the object to be measured, the more gas molecules and suspended particles cluster in the atmosphere, the greater the loss in the phase of infrared transmission, which would also affect the precision of infrared temperature measurement. As a result, when the IRT imager is used to measure the surface temperature of an object, it is required to accurately measure the parameters such as temperature, humidity, and measurement distance (*d*), and input these parameters into the thermal imager to improve measurement accuracy [60].

## 3. Applications of IRT Imaging Technology in Animal Production

In nature, objects higher than absolute zero are able to emit infrared radiation permanently; this effect is known as thermal radiation. The infrared radiation emitted by animals is closely related to the animal body’s metabolic activities, health status, and thermal balance [61]. The animal’s body surface temperature could represent its physiological state. Heat generation and heat release in the bodies of typical organisms are in a state of dynamic equilibrium. Furthermore, total heat generation (including primary metabolic heat output and muscle heat production) equals total heat dissipation (including radiation and evaporation heat dissipation). However, when the normal physiological state of animals is altered, such as the occurrence of early diseases, stress, estrus, or pregnancy, the thermal balance of animals is disrupted, and the body surface temperature of the animal would change dramatically [62]. Infrared imaging technology can quickly capture the temperature changes between the animal body surface and core areas and temperature differences between natural and pathological physiological conditions, which is the basis of early diagnosis [63,64,65]. In recent years, infrared thermography has been increasingly used in animal production [66]. We have reviewed the research literature of recent years for a systematic summary of its application and a detailed overview of the parameters used, such as emissivity, distance, temperature and humidity of the environment in which it is used, as shown in Table 1.

### 3.1. Detection of Animal Surfaces and Core Anatomical Areas’ Temperature Fluctuation

As a traditional temperature measurement method, rectal temperature is still widely used in actual animal production [16,95]. However, the process of rectal temperature measurement would cause discomfort and quickly produce contact stress [96]. IRT imaging technology can dynamically detect the temperature fluctuation of the animal body surface and core areas and analyze the correlation with the rectal temperature, which provides a theoretical basis for the wide application of IRT technology in animal production. In addition, compared with conventional mercury thermometers, temperature measurement by infrared thermography is less likely to cause stress and anxiety and avoid disease transmission caused by contact between thermometers [97]. Tabuaciri [66] used IRT to measure the temperature of piglets, including the base and tips of the ears, and then compared these with the rectum temperature. The results show that the ear base temperature had the most significant correlation with the rectum temperature (R = 0.85), indicating that the ear base could be used as a proxy for these core areas to identity hypothermic piglets via IRT imaging. In addition, Traulsen [98] used infrared imaging technology to measure the temperature of adult sows’ eyes, udder, inner ear, ear root, and vulva, then analyzed the relationship with rectal temperature. He discovered that the udder and vulva have the highest correlation with rectal temperature (R = 0.5), which is expected to become the core area of adult sows’ body surface temperature monitoring. In addition, Vicente-Perez [67] collected infrared temperatures from the head, hip, waist, and abdomen of pregnant ewes exposed to natural heat stress. It found that abdominal temperature accurately reflected changes in rectal temperature, which further indicates that noninvasive infrared temperature measurement could be used to replace the traditional rectal temperature measurement. Furthermore, George et al. [68] measured the infrared temperature of the eyes and muzzles of multiparous ewes. They performed a correlation study with rectal temperature and discovered that ewe eye temperature was strongly correlated with rectal temperature.

**Figure 2:**
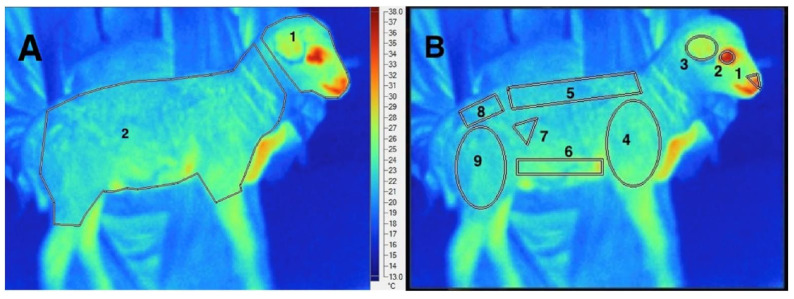
IRT imaging technology in the detection of animal core temperature fluctuation. Infrared thermography from neonatal lambs sectioned in different body sites: (**A**) (entire head (1) and entire body (2)) and (**B**) (muzzle (1), eye (2), ear (3), shoulder (4), loin (5), belly (6), right flank (7), rump (8) and leg (9)). Reprinted with permission from Ref. [71]. Copyright Elsevier and copyright clearance center.

In recent years, IRT imaging technology has also been used to detect the effect of environmental factors on the temperature of the animal body surface and core areas in real-time. For example, Church [69] used thermal imaging technologies to calculate an Angus bull’s eye temperature under various environmental conditions. As compared to rectal temperature association research, the study discovered that the atmosphere of the wind and the sun had an effect on the temperature of the bull’s eye—that when the winds reached 12 km/h, the bull’s eye temperature fluctuated, and when the bull was in the sunshine, the eye temperature increased significantly. Additionally, Peng et al. [70] found that body surface temperatures are more sensitive to environmental conditions than rectal temperatures. The forehead is a relatively reliable area for assessing heat stress responses compared to the eye, ear, cheek, flank, rump, fore udder, and rear udder regions (Figure 2). Additionally, in early spring (predominantly cold) environments, the main cause of neonatal lamb mortality is hypothermia. A recent study pointed out that changes in body surface infrared temperature of wool sheep neonates are more closely related to climatic variables than rectal temperature, allowing effective monitoring of fetal heat loss to prevent lamb mortality [71].

### 3.2. Early Diagnosis of Animal Diseases and Inflammation

The emergence of animal diseases and inflammation usually triggers an increase in local blood circulation and tissue metabolism, resulting a body surface temperature increase and the occurrence of fever [99]. The IRT imager can detect such temperature changes in a non-invasive and non-contact manner. IRT imaging technology is commonly used to detect the occurrence of early cow mastitis. For instance, using IRT imaging technology, Berry et al. [72] detected the diurnal variation of the surface temperature of dairy cows’ udders. They found that the temperature of the infected udder part increased significantly. Additionally, Metzner et al. [73] used thermal imaging technology to diagnose mastitis and discovered that cows with mastitis had an average udder surface temperature that was 2.06 °C higher than that of healthy cows. Furthermore, compared to healthy feet, Anagnostopoulos et al. [100] found that interdigital skin infrared temperature (IST) was high in feet with digital dermatitis (DD) lesions. It suggests the potential for IRT for routine on-farm diagnosis of active DD lesions.

**Figure 3:**
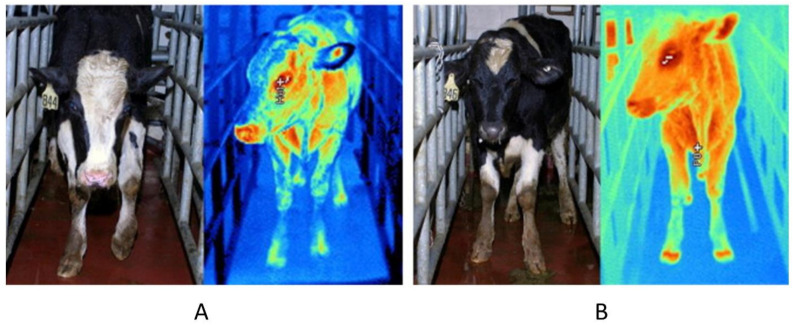
IRT imaging technology in early diagnosis of animal diseases. Infrared images of cattle without (**A**) and with (**B**) fever and viremia at 24 h post challenge, before vesicular lesions were observed. Reprinted with permission from Ref. [75]. Copyright Elsevier and copyright clearance center.

IRT imaging has also been used to diagnose diseases caused by bovine viral diarrhea viruses, respiratory viruses, and foot-and-mouth viruses. For instance, Schaefer et al. [74] found that after infection with bovine viral diarrhea virus, infected animals’ infrared eye temperature increased dramatically in the first few days, indicating that infrared imaging technology can be used for early diagnosis of infected animals. Timsit et al. [101] used IRT imaging technology to dynamically track the rumen temperature of cattle suffering from respiratory diseases. They observed that respiratory infections in cattle would significantly increase rumen temperature. Moreover, Lovett et al. [75] reported that after animals were infected with the foot-and-mouth disease virus, the affected animals’ hoof temperature rose dramatically before clinical signs occurred, which could be detected sensitively by IRT imaging, contributing to the early diagnosis and treatment of the disease (Figure 3).

IRT imaging technique is also commonly used in equine medicine and the early identification of illnesses in pigs, such as clinical diagnosis of equine hooves and pig lameness. Eddy et al. [76] discovered flexor tendons of horses that were already injured and inflamed before clinical claudication, because the temperature in the tendon’s local area rose; IRT imaging technology could be used to identify and diagnose the condition effectively. Lameness [102] seriously affects the daily activities of pigs. Amezcua et al. found that IRT imaging technology could be used to detect lameness in the lower limbs of pigs as an effective means for early diagnosis of claudication. In addition, Siewert et al. [77] used an IRT imager to collect the average body surface temperature of the core region of pigs to detect fever (>39.5 °C). The specificity and sensitivity of the infrared detection results were as high as 85% and 86%, indicating that an IRT imager can be used to diagnose fever in pigs.

### 3.3. Detection of Animal Stress Level

Physiological parameters such as body temperature and respiration rate are used to assess animals’ adaptability and tolerance to their surroundings. However, measuring these parameters usually necessitates the restraint of animals, which can easily result in the stress response of the animals [103]. As a noninvasive and remote monitoring technology, IRT imaging technology can effectively help identify animal stress. An IRT imager shows elevated body temperature due to changes in blood flow caused by stressful environmental conditions. Thus, farm animals’ physiological parameters and stress levels can be predicted by using infrared temperatures in specific areas of the animal, such as the eyes, muzzle, abdomen, and udder [78,79,104]. For instance, Weschenfelder [104] used infrared imager to measure the eye temperature of pigs before slaughter and discovered that it was positively correlated with blood lactic acid level, pH, and longissimus muscle drip loss. It indicates that IRT imaging technology is expected to become an essential tool for assessing pig stress levels prior to slaughter and predicting pig quality after slaughter. Additionally, Tangorra et al. [80] found that mechanical stress on cow teats caused by milking machines changed their color from pink to red or purple. The infrared temperatures at the base, middle, and tip of the teat increased significantly at this time, suggesting that infrared thermography can be used to detect short-term levels of mechanical stress on teats caused by milking machines.

Paim et al. [81] discovered that the infrared temperature of lambs’ mouths, necks, and buttocks could be used as a good indicator of their environment’s thermal comfort and to evaluate the thermal status and stress level of lambs. Valera et al. [82] used a thermal imager to measure the maximum eye temperature of racehorses and compared it to the cortisol level in their saliva. They discovered that IRT imaging technology could effectively evaluate racehorse stress during the race. Stewart [15] also confirmed that the infrared eye temperature of cows could be used as an essential index to assess stress. Similarly, Abuabos et al. [83] employed IRT imaging technology to evaluate the effects of stocking density on broiler physiological stress response. The results show that the head, neck, wings, and tibia of medium-density and high-density breeding chickens had a higher IRT temperature when compared to low density. Increased stocking density would be detrimental to animal welfare. Additionally, Narayan et al. [84] used IRT imaging technology to detect the core temperature of koalas on their body surface. They discovered that the infrared weather of the lacrimal caruncle in koalas’ eyes was the most stable and could best reflect the external characteristics of koalas’ thermal bias. In blue tits (Cyanistes Caeruleus) and cattle stress studies, lacrimal caruncle infrared temperature was also used to assess physiological stress [15,85].

### 3.4. Early Identification of Estrus and Ovulation in Animals

During the estrus cycle, the animals’ body temperature fluctuates dynamically, dropping two days before estrus and then increasing as the expression of luteinizing hormone increases [86]. Temperature changes in the estrus cycle have been associated with luteinizing surge and ovulation. The estrus statement can be calculated using conventional rectal thermometry. However, traditional rectal temperature calculation is time-consuming and induces extreme pain and physiological stress in animals. At present, as a noninvasive monitoring technology, IRT imaging technology is being used to measure the body surface temperature of the animal estrus period to identify the animal estrus. Talukder et al. [87] used IRT imaging technology to detect the temperature of the vulva and nasal mirror of cows to determine the estrus phase of cows, and the findings reveal that infrared technology is more sensitive than visual observation. Freitas et al. [88] used an IRT imager to calculate the core parts of the body surface of ewes in the estrus cycle, including the anus, vulva, and ear. They comprehensively considered the temperature and humidity of the environment and the temperature of the wet bulb and other variables. As a result, IRT technology can detect small temperature changes at different stages of ewe’s estrus cycle and efficiently identify ewes in the early estrus stage. Scolari et al. [89] collected the vulva skin temperature of sows using IRT imaging technology. They discovered significant changes in vulva temperature during ovulation, proving the feasibility of using IRT imaging to predict sow ovulation. The team then used IRT imaging technology to measure sow vulva temperature and discovered that their vulva epidermal temperature changed significantly when they were in estrus.

**Figure 4:**
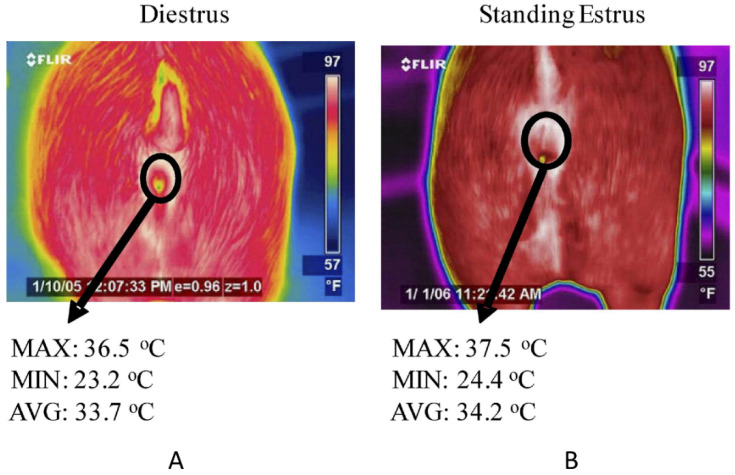
IRT imaging technology in early identification of estrus in animals. The region of interest from which vulva thermal measurements were determined in gilts is indicated by the circle and the maximum (MAX), minimum (MIN), and average (AVG) temperature values of representative gilts (**A**) in diestrus and (**B**) in standing estrus are shown for the thermal images above. Reprinted with permission from Ref. [90]. Copyright Elsevier and copyright clearance center.

Furthermore, Sykes et al. [90] obtained blood samples from sows to reliably assess their estrus cycle stage and then used IRT imaging technology to detect the rectal temperature of the sows. The research discovered that the maximum and average vulva temperature values during the estrus cycle were higher than those during the estrus period (Figure 4). Simoes [91] measured the vulva and buttocks temperature of pigs in estrus and compared it to the non-estrus infrared temperature. The vulva and buttocks temperature was substantially higher, which could be used to predict sow ovulation.

### 3.5. Pregnancy Diagnosis of Animals

The trial procedure, ultrasonic diagnosis, and immunological diagnosis are the most conventional methods of pregnancy diagnosis. However, they are easily influenced by both environmental and individual influences, and the accuracy of diagnosis results is limited. Ultrasound diagnosis and immunological diagnosis are easy to cause animal stress. The temperature of an animal’s main body increases once it becomes pregnant, which can be reliably detected by infrared thermography. At present, IRT imaging technology is increasingly being used in pregnancy diagnosis. Hilsberg [105,106] was the first to use IRT imaging to detect pregnancy in giraffes. Subsequently, they measured the abdominal temperature of pregnant black rhinoceros and grey spotted horses. They discovered that their stomach temperature had dramatically increased.

**Figure 5:**
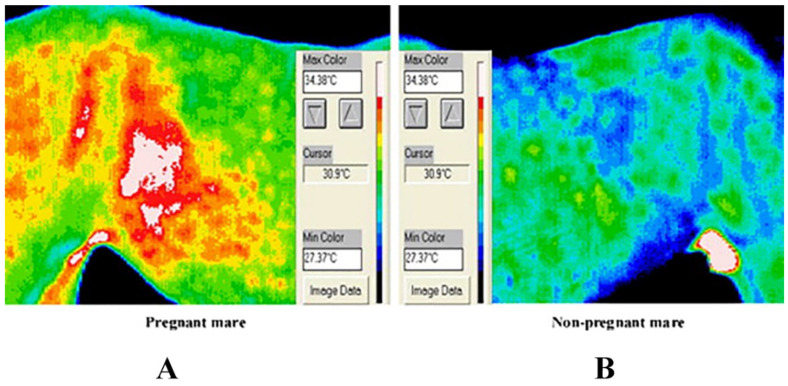
IRT imaging technology in pregnancy diagnosis of animals. (**A**), Infrared image of a pregnant mare. (**B**), Infrared image of a nonpregnant mare. The pregnant mare has a higher flank/abdomen temperature than the nonpregnant mare. Reprinted with permission from Ref. [50]. Copyright Elsevier and copyright clearance center.

Additionally, Durrant [92] used IRT imaging equipment to successfully diagnose panda pregnancy, which is critical for saving endangered animals. Bowers et al. [50] used IRT imaging technology to measure the temperature of pregnant mares’ abdomen and scapula in the late gestation period. As a result, they discovered significant temperature variations between pregnant and nonpregnant mares’ abdomens. This temperature difference was caused by a rise in local metabolic demand caused by fetal growth in the uterus, which resulted in increased blood flow and temperature (Figure 5). Recently, Maśko [93] found that thermal imaging in the flank area of pregnant mares is more suitable for pregnancy diagnosis due to the seasonal fluctuation of hair length. Still, there is no obvious evidence that hair coat features of pregnant horses are related to abdominal temperature.

However, Jones et al. [107] used IRT imaging technology to take the temperature of a pregnant cow’s left abdomen. They discovered no noticeable variation in temperature between pregnant and nonpregnant cows. This was because cows’ rumens became larger and more physiologically active, resulting in higher abdominal temperature, which obscured temperature fluctuations caused by pregnancy. While giraffes are ruminants, their rumen is small compared to that of other ruminants and does not conceal the temperature differential caused by pregnancy. As a result, IRT imaging technology is not appropriate for detecting pregnancy in various mammals, as it is particularly suitable for monoculture animals or animals with a small rumen.

### 3.6. Animals Welfare

**Figure 6:**
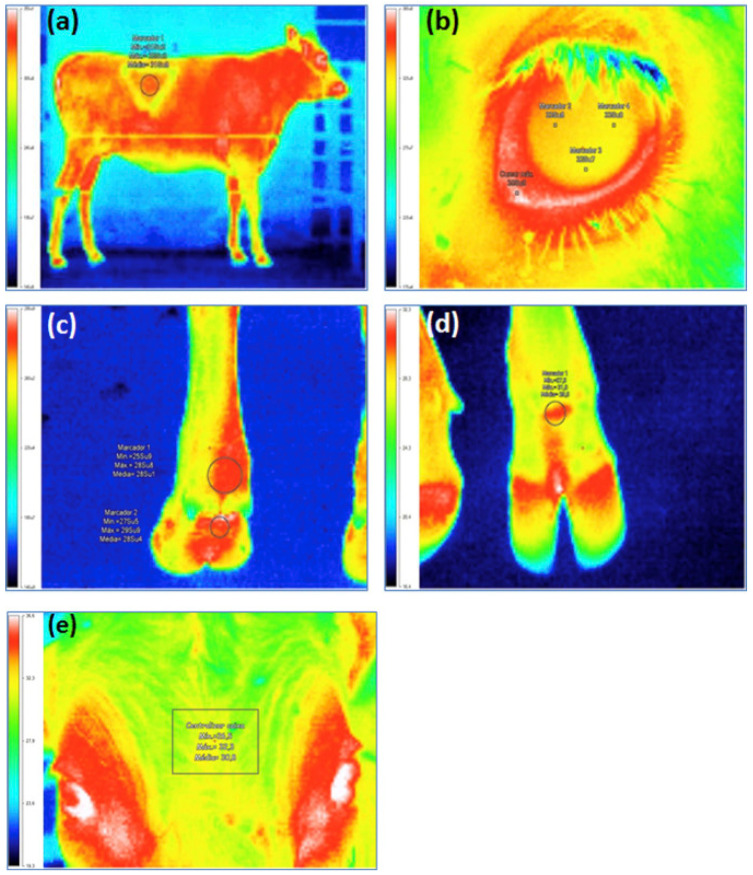
IRT imaging technology in animals welfare. Study regions of infrared thermography for (**a**) flank; (**b**) eye; (**c**) dorsal left foreleg; (**d**) cranial left foreleg; and (**e**) forehead of heifers. Reprinted with permission from Ref. [94]. Copyright Elsevier and copyright clearance center.

Animal welfare has been a global issue for the past few decades. The World Society for the Protection of Animals (WSPA) measures the level of animal welfare in terms of five dimensions: animals are free from hunger, thirst and malnutrition, animals are free from environmental discomfort, animals are free from disease and injury, animals are free from fear and grief, and animals are free to express their nature in appropriate conditions [108]. In intensive animal production, many factors, such as stocking density, environmental deterioration, unsuitable social environment, heat stress, pain, or difficulty in obtaining necessary resources, can be significant sources of stress, leading to a decline in welfare [94,109]. Therefore, assessing the physiological state of fast animals and how to ensure their welfare has become an important research topic in the last 20 years. IRT technology has been widely used in animal welfare assessment by identifying changes in the temperature of animal body parts and monitoring the physiological status of animals. For instance, Salles [94] used IRT to measure body surface temperature of Jersey cattle in a thermoneutral environment (see Figure 6). They found that forehead temperature had the highest correlation with rectal temperature, which could be used for future thermoregulation, body heat production, and animal welfare studies. In addition, hypothermia is a risk factor for neonatal mortality in piglets, especially in low-birth-weight piglets. Piglets with intrauterine growth retardation (IUGR) are also at a higher risk of death at birth. Schmitt et al. [38] found the strongest correlation between the infrared temperature at the base of the ear of piglets and their rectal temperature, which can be used to rapidly and dynamically monitor piglet body temperature and assess the thermoregulatory capacity of newborn piglets. It suggests that IRT can implement interventions for piglets at risk of hypothermia and can further improve animal welfare.

## 4. Conclusions and Prospect

As an essential physiological index, animal body surface temperature can be used to accurately evaluate the physiological state of animals under stress, fertility, welfare, metabolism, health, and disease. Real-time and rapid detection of animal surface temperature is significant for animal production. The application of IRT technology in animal production is innovative. IRT has high-temperature sensitivity and spatial resolution, uses a non-contact method, and can quickly and efficiently collect animal surface temperature without direct physical contact with animals. In this paper, we systematically reviewed the application of IRT technology in all aspects of animal production and analyzed the basic principles of infrared thermal imaging and factors affecting infrared thermal imaging to help researchers in related fields better master this technology.

At present, IRT technology still has some shortcomings. Firstly, the infrared thermal imaging device highly depends on selecting sensors and experimental settings. The hardware equipment is relatively expensive, requiring higher operators and professional training requirements. Due to a lack of funds, it is challenging to promote the application of small and medium-sized ranches. Secondly, IRT technology is easily affected by environmental factors. The pasture’s complex ecological factors will affect temperature measurement accuracy to a certain extent. Of course, technical personnel can set parameters to reduce errors. Finally, infrared thermal imaging is very sensitive in detecting temperature changes in animals. However, sometimes this method cannot determine the direct cause of temperature differences and still requires traditional diagnostic methods. In the future, with the continuous improvement of IRT technology and detection sensitivity, it will be endowed with new uses and definitions. Therefore, IRT technology will be used more and more in animal production.

## Figures and Tables

**Table 1:** Applications of IRT imaging technology in animal production.

Animals	Type of IR Camera	Emissivity	Distance	Environment Temperature	Humidity	Application	References
Piglet	FLIR	0.98				Detecting animal surfaces and core anatomical areas temperature fluctuation	[66]
Ewe	Fluke Ti10			25.7–42.0 °C	42%		[67]
Ewe	FLIR P65HS		30 cm	25.6–34.0 °C	81.3% ± 9.4%		[68]
Cattle	FLIR I40 and E60	0.95	1.0 m				[69]
Dairy cow	InfraTec		1.5 m				[70]
neonatal lamb	Fluke Ti10		1.0 m	10.4–34 °C	7.9–100%		[71]
Dairy cow	FLIR 760		2.0–2.5 m	11.1–27.4 °C		Diagnosing animal early diseases and inflammation	[72]
Dairy cattle	FLIR B320	0.96	1.8 m				[73]
Heifer	FLIR 760		1–3 m	14 °C	28%		[74]
Cattle	FLIR EX320		1.5–2.0 m				[75]
Sow	FLIR T300		0.5–0.8 m				[76]
Pig	FLUKE R2	0.97	30–50 cm	21.1–22.6 °C			[77]
Dairy cattle	FLIR SC2000	0.98	1.5 m	11.93–29.04 °C	26.09–79.13%	Detecting animal stress level	[78]
Pig	FLIR SC660	0.98	50 cm	16.2–24.3 °C			[79]
Cow	GEAR-G120	0.98	0.5 m	15–25 °C	67–78%		[80]
Lamb	FLIR series	0.95					[81]
Horse	FLIR I700		1 m	16–31 °C	56–68%		[82]
Broiler chicken	VisIR-Ti200	0.97		24.73–25.18 °C	23.04–34.43%		[83]
Koala	FLIR 530		1 m				[84]
Bird		0.97	40–500 mm				[85]
Dairy cow	FLIR A310	0.98	1 m	14.05 ± 3.06 °C	68.86% ± 6.94%	Diagnosing animal estrus and ovulation	[86]
Cow	FLIR 620	0.98	1 m				[87]
Ewe	FLIR I50	0.98	1 m	26.77–31.01 °C	64.74–83.62%		[88]
Swine	Fluke		0.61 m				[89]
Gilt	FLIR S60	1	121.9–152.4 cm	1.5–25.8 °C			[90]
Sow	Fluke 9 HZ		1 m				[91]
Giant panda	FLIR PM545		0.6–0.9 m			Diagnosing animal pregnancy	[92]
Mare	Compix vet2000	0.96	147.3–157.5 cm	4.2–28.9 °C			[50]
Mare	FLIR E60	0.99	2.0 m	10–40 °C			[93]
Piglet	FLIR T420	0.98	1 m			animals’ welfare	[38]
Cattle	Fluke Ti20TM	0.98	20 cm–2 m	17.7–27.3 °C	59.3–90.9%		[94]

Abbreviations: Blank part of the table: no relevant parameter information.

## Data Availability

Not applicable.
